# The Influence of Gut Microbiota on Neurogenesis: Evidence and Hopes

**DOI:** 10.3390/cells11030382

**Published:** 2022-01-23

**Authors:** Fiorella Sarubbo, Virve Cavallucci, Giovambattista Pani

**Affiliations:** 1 Faculty of Science, University of the Balearic Islands UIB, 07122 Palma, Spain; fiorella.sarubbo@uib.es; 2 Research Unit, Son Llàtzer University Hospital, Health Research Institute of the Balearic Islands (IdISBa), 07198 Palma, Spain; 3 Fondazione Policlinico Universitario A. Gemelli IRCCS, 00168 Rome, Italy; 4 Institute of General Pathology, Università Cattolica del Sacro Cuore, 00168 Rome, Italy

**Keywords:** gut microbiota, gut-brain axis, adult neurogenesis, ageing, neural stem cells, neurodegeneration, nutrients, antioxidants, polyphenols

## Abstract

Adult neurogenesis (i.e., the life-long generation of new neurons from undifferentiated neuronal precursors in the adult brain) may contribute to brain repair after damage, and participates in plasticity-related processes including memory, cognition, mood and sensory functions. Among the many intrinsic (oxidative stress, inflammation, and ageing), and extrinsic (environmental pollution, lifestyle, and diet) factors deemed to impact neurogenesis, significant attention has been recently attracted by the myriad of saprophytic microorganismal communities inhabiting the intestinal ecosystem and collectively referred to as the gut microbiota. A growing body of evidence, mainly from animal studies, reveal the influence of microbiota and its disease-associated imbalances on neural stem cell proliferative and differentiative activities in brain neurogenic niches. On the other hand, the long-claimed pro-neurogenic activity of natural dietary compounds endowed with antioxidants and anti-inflammatory properties (such as polyphenols, polyunsaturated fatty acids, or pro/prebiotics) may be mediated, at least in part, by their action on the intestinal microflora. The purpose of this review is to summarise the available information regarding the influence of the gut microbiota on neurogenesis, analyse the possible underlying mechanisms, and discuss the potential implications of this emerging knowledge for the fight against neurodegeneration and brain ageing.

## 1. Introduction

The search for new therapeutic targets against brain ageing and associated neurodegenerative diseases represents one of the most urgent and challenging issues in current biomedicine, due to the increasing proportion of the elderly population worldwide [1]. Among the leading causes of ageing and neurodegeneration, the limited renewal capacity of brain cells [2], alterations of brain vasculature [3,4] and neuronal/glial dysfunction [5] are accompanied by an age-dependent decline in the brain damage repair systems, which include adult neurogenesis [6,7,8,9,10]. Neurogenesis can be defined as the generation of new neurons, glial cells and other neural lineages from neural stem cells (NSCs) and neural progenitor cells (NPCs) [11,12]. This process includes the maturation, migration and functional integration of NSCs or NPSs into the preexisting neuronal network [13,14]. When it occurs in adult life, it is known as adult neurogenesis (AN). Although NSCs are present in several brain regions, the subgranular zone of the hippocampus and the subventricular zone of the lateral ventricle are the main AN niches [15]. AN in other adult brain regions (e.g., the neocortex, striatum, amygdala and substantia nigra) is limited under normal physiological conditions, but could be induced after injury [16]. Maintenance of neurogenesis contributes to brain repair after damage and is believed to play a role in stress-responses and higher functions involving brain plasticity such as memory and cognition [6,17,18,19,20], mood [21], or perceptual (e.g., olfactory) learning [22,23]. Accordingly, an impairment in neurogenesis, as seen during ageing or in pathological conditions [24], has been associated with seizures [25,26], depression [27], and decline of learning abilities [28]. Impaired neurogenesis may occur because of a reduction in the number and/or function of NSCs and NPCs [29]. This may be due to the synergic action of several mechanisms operating in the brain in ageing or neurodegenerative conditions: inflammation [30,31], oxidative stress [32], or toxic substances like short-chain fatty acids (SCFAs), branched chain amino acids and peptidoglycans, originating from an altered intestinal microbiota [33]. Gut-resident microbial communities are in turn modulated by extrinsic factors, such as lifestyle and diet; importantly, imbalances affecting this complex ecosystem can impact the permeability of the body barriers, including the blood brain barrier (BBB) and the enteric barrier, so as to allow the passage of potentially noxious substances to brain tissue along the so-called gut-brain axis (GBA) [34,35].

To counteract the deterioration of neurogenesis, mechanisms that could be exogenously regulated, such as the composition of gut microbiota, are of particular interest. Gut microbiota is comprised of several species of microorganisms, including bacteria, yeast, and viruses [36], cohabiting in a delicate balance whose disruption (dysbiosis) can lead to aberrant neural and glial reactivity accompanied by loss of neurogenic ability [37]. Thus, a functional relationship links microbiota, GBA and neurogenesis [5,34], and alterations in this axis not only affect the neural regulation of the gastrointestinal tract, but, also contribute to several brain disturbances, such as mood (e.g., depression, anxiety) and neurodevelopmental (e.g., autism) [38,39] and cognitive disorders (e.g., Alzheimer’s disease) [34,40,41,42]. Therefore, in establishing a bidirectional connection between enteric microbes and the brain, GBA exploits several anatomic structures, systems, and metabolic routes [34], such as the neuroendocrine (by the hypothalamic–pituitary–adrenal (HPA) axis) and neuroimmune systems, the sympathetic and parasympathetic arms of the autonomic nervous system, including the enteric nervous system, the vagus nerve [43], and the immune system. Not surprisingly, therefore, the GBA has been portrayed as a “second brain” [34].

As an additional layer of complexity, many factors can influence microbiota composition, including infection, mode of birth delivery, use of antibiotic medications, the nature of nutritional provision, environmental stressors, host genetics and ageing [44,45]. Among the potential therapeutic approaches aimed at the microbiota to target GBA and neurogenesis, diet composition appears particularly attractive for its feasibility. For instance, natural antioxidants and anti-inflammatory molecules, such as dietary polyphenols, have long been investigated as potential adjuvants to support AN [46]. In simple terms, maintaining a healthy brain across the lifespan [47] may simply require “good” intestinal bacteria and the right diet to keep them going.

The purpose of this review is to summarise the currently available information regarding the influence of the gut microbiota on AN and the potential of microbiota-centred interventions as a strategy against brain ageing and neurodegeneration. To address the topic we followed the classical methodological frameworks for a state-of-the-art review [48]. A list of keywords (neurogenesis, ageing, neural stem cells, gut-microbiota, gut-brain-axis, nutrients, polyphenols, and neurodegeneration) was initially identified. Then, different keyword combinations, each containing the term “neurogenesis”, were used to interrogate the following sources: PubMed, Embase, Medline, Scopus, Web of Knowledge and Google Scholar. Articles published in English and indexed as original articles, meta-analysis reviews, narrative reviews, clinical cases and comments to the editor, with qualitative and quantitative data, were included in the analysis. Although the time range was not limited, the most recent publications were prioritized.

## 2. Evidence for the Connection between Intestinal Microbiota and Neurogenesis

**Figure 1:**
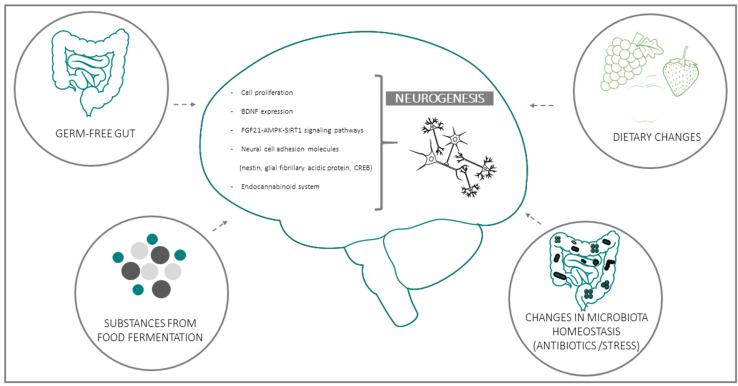
Schematic representation of the four main experimental models used to investigate the functional linkages between intestinal bacteria and adult (mainly hippocampal) neurogenesis. Biochemical and functional parameters employed in most studies for the evaluation of neurogenesis and its microbiota-induced modifications are listed in the central, brain-shaped field.

Several clinical and experimental studies point to a functional connection between intestinal microbiota and neurogenesis through the GBA. This emerging evidence implies that microbiota composition may represent both a causative determinant and a therapeutic target in diseases where neurogenesis plays a key role [47,49,50,51,52]. Experimental data in support of the influence of microbiota on AN can be grouped in four general domains: (a) data from Germ-free (GF) animals; (b) data on substances derived from bacterial fermentation of food; (c) changes in bacteria homeostasis due to exogenous factors (e.g., antibiotics or stress); (d) consequences of dietary changes (Figure 1):

(a) GF gut: GF animal models, usually mice or rats, grown up without any exposure to microorganisms, constitute an essential tool in studying the influence of the gut microbiota on brain function; not surprisingly, one of the first studies that highlighted the effect of the microbiota on neurogenesis was conducted on this model.

Using bromo-2-deoxyuridine (BrdU) immunohistochemistry, it was shown that, compared to conventionally raised mice, GF and GF–colonized mice exhibited a trend to increased cell proliferation, predominantly in the dorsal hippocampus, accompanied by alterations in the hippocampal brain-derived neurotrophic factor (BDNF) [19]. In agreement, another study reported an altered expression of synaptic plasticity-related genes, with significantly lower BDNF mRNA expression in the hippocampus, amygdala, and cingulate cortex in GF mice; of note, these areas participate in neurogenesis and are key components of the neural circuitry underlying behaviour. Along similar lines, Kundu and colleagues [49], investigated the effects of transplanting the gut microbiota from young or old donor mice into young GF recipient mice [49]. They found that the transplant-induced hippocampal AN is in parallel with the activation of the pro-neurogenic FGF21-AMPK-SIRT1 signalling pathway. Moreover, it has been observed that intestinal bacteria and components of the bacterial cell wall maintain the adult enteric neuron system and nitrergic neurons by promoting intestinal neurogenesis via the Toll-like Receptor 2 (TLR2) [53].

(b) Substances produced by food fermentation: Converging lines of evidence point to the potential role of food fermentation substances produced by gut bacteria on the modulation of AN. This is the case of the SCFA butyrate, synthesized from non-absorbed carbohydrates by colonic microbiota [54]. In an animal model of ischemia it was demonstrated that the histone deacetylase inhibitor, sodium butyrate, stimulates the incorporation of BrdU in the subgranular and the subventricular zone of the hippocampus, striatum, and frontal cortex in rats subjected to permanent cerebral ischemia. This treatment also increased the number of cells expressing the polysialic acid-neural cell adhesion molecule, nestin, the glial fibrillary acidic protein, the phospho-cAMP response element-binding protein (CREB), and BDNF in various brain regions after brain ischemia [55]. Accordingly, it was also demonstrated that oral sodium butyrate impacts brain metabolism and hippocampal neurogenesis in pigs [56].

(c) Changes in bacteria homeostasis due to exogenous factors (e.g., antibiotics or stress): Prompted by the emerging notion that the intestinal ecosystem can influence the vegetative and cognitive functions of the host [57,58], several studies have focused on the impact of antibiotics on microbiota and gut-brain communication. In mice, depleting gut microbiota with antibiotics, from weaning onward, induces cognitive deficits, specifically in memory retention, and leads to a significant reduction of BDNF in the adult brain, maybe by the involvement in AN [59]. However, while consistent with the observed changes, a specific impact of microbiota depletion on neurogenesis was not directly demonstrated in this study. This aspect was instead specifically addressed by Môhle et al. [60], who reported a long-lasting impairment in neurogenesis, accompanied by behaviour deficits in antibiotic-treated mice. It is worthy of note that these alterations were partially restored by exercise (running) and probiotics administration. Mechanistically, the above treatments increased the number of Ly6C(hi) monocytes [60], a cell population involved in immune surveillance and host defense upon infections and inflammation. Moreover, elimination of Ly6Chi monocytes by antibody depletion or by using knockout mice resulted in decreased neurogenesis, whereas the adoptive transfer of Ly6Chi monocytes was able to preserve neurogenesis after antibiotic treatment [60].

Besides antibiotics, the homeostasis of intestinal microbiota can also be affected by other drugs and stress factors. Chronic stress can impact gut microbiota diversity, promoting an increase in pathogenic bacteria at the expense of beneficial ones (dysbiosis). This imbalance, in turn, affects lipid metabolism and decreases the endocannabinoid signalling system, thus reducing hippocampal AN. Of note, dysbiosis frequently accompanies ageing and may lead to chronic inflammation and a decrease in pro-neurogenic bacterial metabolites (such as SCFAs) in the senescent intestine [61].

(d) Dietary changes: High-fat or choline-deficient diets produce a specific gut microbiota signature in the small intestine and cecum, marked by increased propionate and butyrate synthesis, mitochondrial biogenesis and generation of reactive oxidative species (ROS) downstream of SCFAs. All of these variations affect NSCs fate, leading to premature differentiation and depletion of the NSC pool in the AN niches of high-fat or choline-deficient-fed mice, ultimately impairing AN [47]. On the other hand, dietary or probiotic interventions have been indicated as effective therapeutic approaches to fight stress-associated neurological disturbances operating through the GBA [17]. Importantly, a clinical study on bacterial strains known to boost neurogenesis in mice reported improved cognitive functions in adult patients with major depression; while the involvement of neurogenesis in the effects observed in human subjects can be only indirectly inferred; the consistency with results gleaned in the preclinical setting is intriguing [62,63]. Furthermore, with regard to probiotics, it was found that in a rat model of early-life stress, maternal separation caused a marked decrease in hippocampal BDNF, while the probiotic *Bifidobacterium breve 6330* increased BDNF to levels observed in control animals, suggesting that BDNF might be involved in the regulation of anxiety through microbiome-GBA [64]. Thus, diet and probiotics represent major environmental determinants of the gut flora composition [65] and, as such, constitute potential tools for the restoration and maintenance of brain homeostasis. Further information regarding extrinsic modulators of neurogenesis is found Section 3.2.

### GBA: Physiological Architecture of the Communication Way between the Intestinal Microbiota and the Brain

**Figure 2:**
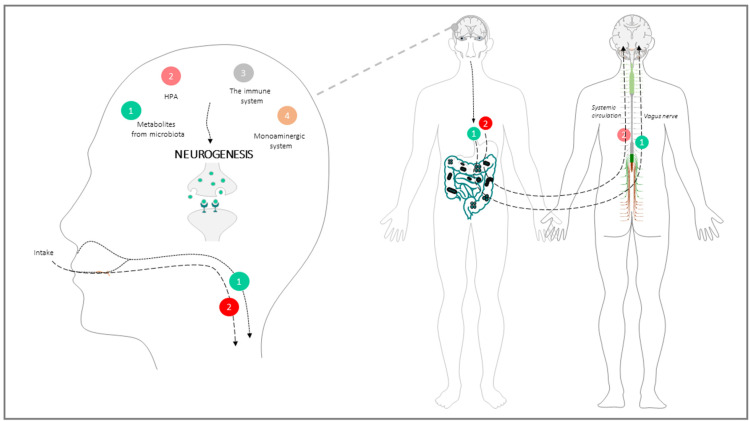
Anatomic-functional structure of the GBA. Dietary factors modify microbiota composition and metabolism leading to the formation of secondary compounds that signal to the brain. Microbial inputs are either relayed by the vagus nerve afferent fibers (green route) or travel through the systemic circulation and the BBB to the SNC, where they perturb the HPA (red route). Vagus nerve afferences and HPA, together with signals from the monoaminergic system and immune/inflammatory molecules modify neurogenic circuits. More details regarding the brain-gut limb of the axis are in the main text.

The communication between the gut microbiota and brain through the GBA is the result of a long-term symbiosis and co-evolution process which involves immunological, endocrine, neurological, and metabolic signalling pathways [37]. The physiological mechanisms and elements underlying this neurogenesis-impacting communication involve: (a) The parasympathetic system, mainly the vagus nerve; (b) The monoaminergic system; (c) The neuroendocrine system, mainly the HPA; (d) The immune system; (e) biochemical metabolites from microbiota metabolism (Figure 2).

(a) The parasympathetic system: Neural transmission through the vagus nerve can be activated or inactivated by microbial factors synthesized in the intestine, and represents the main communication route between gut and brain. For instance, SCFAs produced during food fermentation can evoke CNS responses by activating vagal chemoreceptors. One remarkable example of these central responses is the “inflammatory reflex”, whereby efferent inputs travelling through the vagus nerve inhibit the release of pro-inflammatory cytokines in the periphery. Anti-inflammatory signals in turn preserve intestinal barrier integrity, and, by doing so, indirectly affect hippocampal plasticity and neurogenesis [66]. On the afferent side of the vagus nerve-mediated communication, microbial signals may have a role in supporting cell growth, differentiation and survival during neural development [67]. In fact, vagotomized mice displayed a decrease of BDNF mRNA in all areas of the hippocampus together with a reduced proliferation and survival of newborn cells, and a decreased number of immature neurons, especially those with complex dendritic morphology [68]. Thus, gut microbiota may modulate brain BDNF expression (and by extension, AN) through neuronal inputs relayed by the vagus nerve. Of note, altered BDNF expression is a distinctive feature of several neurodegenerative disorders, e.g., Alzheimer’s disease [69].

(b) The monoaminergic system: Neural-mediated effects of the gut microbiome on hippocampal neurogenesis may also involve the monoaminergic (especially serotoninergic) system [37]. Serotonin and its precursor tryptophan are important signalling molecules in both the CNS and the gastrointestinal tract. Serotonin exerts modulatory effects on stress, anxiety, mood, and cognition [70,71]; moreover, it participates in hippocampal homeostasis and promotes hippocampal AN [72]. Investigating the impact of intestinal microbiota on the hippocampal serotonergic system, Agus et al. 2018 reviewed this topic and explain that in several experiments, compared to conventionally raised controls, GF male mice had elevated serotonin levels and increased plasma concentrations of tryptophan [73]. The authors also found that fecal transfer from mice exposed to mild chronic stress to healthy controls impaired the pro-neurogenic effects of fluoxetine, a standard selective serotonin reuptake inhibitor previously known to promote the proliferation, differentiation, and survival of progenitor cells in the hippocampus, and to influence the plasticity of newly generated neurons [74].

(c) The HPA axis: Evidence exists that metabolites released by intestinal microbes may enter the bloodstream, cross the BBB and directly reach the brain, where they cause hormonal interference as well as cognitive changes [75]. Perturbation of the HPA axis in turn results in intestinal dysfunction via excess release of glucocorticoids (cortisol and corticosterone), mineralocorticoids (aldosterone) and catecholamines (dopamine, epinephrine and noradrenaline) [76]. The impact on HPA and the neuroendocrine axis likely represents a major mechanism for microbiota-related brain and behavioural changes [75].

Neural stem and progenitor cells (NSPC) express the glucocorticoid and mineralcorticoid receptors, and several in vitro studies have highlighted a direct inhibitory effect of corticosteroids (especially at high doses) on the proliferative and differentiative capacity of neural precursors [77,78]. Mechanistically, dexamethasone, via the glucocorticoid receptor, induces the expression of DKK1 (Dikkopf1), an inihibitor of neurogenic signalling through the Wnt pathway, in human NPSC [79]. Additionally, high dose cortisol, possibly through the serum and glucocorticoid inducible kinase 1, inhibited Hedghog signalling in human hippocampal precursor cells, while downstream reactivation of the same pathway by the *smoothened* agonist purmorphamine cancelled the inhibitory effect of cortisol on neuronal differentiation [78]. Also of note, as in other cell types, cortisol elicits neural progenitor resistance to insulin/insulin-like growth factor (IGF) signalling and blunts the activation of the major downstream effectors ERK1 and AkT-mTOR. Accordingly, high dose IGF1 reversed the differentiation and survival defect displayed by cortisol-exposed rat embryonic NSC [80]. Given the relevance of the above pathways for (adult and embryonic) neurogenesis, and their deep interconnection (i.e., Akt and Wnt signalling converge on the inhibition of GSK3 beta), it is conceivable that GBA-controlled neurormonal stress responses impact directly on niche-derived signals and the downstream intracellular pathways that normally govern neurogenesis.

(d) The immune system: microbes govern the induction, training and function of the host immune system. Reciprocally, the immune system maintains the symbiotic relationship of the host with the biological diversity present in the microbiome. When operating optimally, the immune system-microbiota alliance allows the induction of protective responses to pathogens and the maintenance of tolerance to innocuous antigens [81].

The microglia constitute the most abundant innate immune cell population of the CNS; microglial cells belong to the macrophage lineage, and comprise between 10% and a 15% of all glial cells. Microglia is involved in CNS homeostasis, antigen presentation, phagocytosis, and control of inflammation throughout life [82]. A recent study provided direct evidence that microbiota can influence neurogenesis by modulating the brain immune system specifically through this cell population [83]. In weaning mice administered a low dose of dextran sodium sulphate to induce acute colonic inflammation, Salvo et al. 2020 found that alterations of intestinal bacterial populations were paralleled by behavioural deficits, diminished neurogenesis in adulthood, and increased hippocampal expression of genes encoding pattern recognition receptor and T-helper 17 cell-related cytokines. Moreover, hippocampal microglia displayed an activated phenotype in these animals, as revealed by increased expression of the gene encoding the ionized calcium-binding adapter molecule 1(Iba1) [83]. Bacterial-derived SCFAs have been shown to have a key role in microglial maturation and its efficient functioning. GF mice display a reduction in both microglial maturity and number, with morphological microglia abnormalities compared to control mice. Likewise, mice treated with antibiotics show decreased microglial maturity [84]. Furthermore, the microglia of GF mice do not exhibit an activated phenotype in response to the intrusion of bacteria and viruses, which highlights the critical role of microbiota in mounting an appropriate immune response in the CNS. Additionally, the immature phenotype of microglia was also observed following antibiotic-induced microbiota depletion in specific pathogen-free mice, albeit with no significant change in the cell number [85]. Importantly, the administration of SCFAs, food fermentation products of microbial metabolism, normalized microglia functions in GF mice, indicating that the gut microbiota is essential for the normal structure and function of this unique immune cell population.

Besides microglia, astrocytes also participate in the CNS-microbiota cross-talk. Astrocytes have significant roles in ion homeostasis, neurotransmitter clearance, maintenance of the BBB, support of neuronal signalling and relevant to this article, protection against neuroinflammation [86].

Finally, as an additional line of immune/inflammatory communication between gut microbes and the brain, evidence exists that bacteria and immunoregulatory factors released from peripheral sites under the influence of the microbiome can damage the BBB and alter its physical integrity and transport selectivity, or induce the local release of neuroimmune substances from the barrier cells, ultimately leading to mental disturbances [86] in a fashion that does not directly involve resident immune cells.

NPCs express receptors for a wide range of cytokines/chemokines, and the establishment of a pro-inflammatory environment in NSC niches is clearly detrimental towards neurogenic activities [87]. Accordingly, by transcriptomic profiling of human subependymal zone (SEZ, a major adult neurogenic area) post-mortem tissue samples of a wide age range, Bitar and colleagues recently reported a marked age-dependent increase of the inflammatory signature, in parallel with a decrease of neurogenesis-related profiles [88]. Moreover, 3D mixed cultures of temporal lobe biopsies from epileptic patients revealed enrichment of inflammatory cells and elevated levels of IL-1 and HMGBI (high mobility group box 1), concomitant with impaired neurogenesis in vitro. Interestingly, the pharmacological blockade of the two cytokines significantly improved precursor proliferation and differentiation in 2D and 3D cultures, consistent with the notion that inflammatory signals impair neurogenesis [89]. While the effect of inflammation and related oxidative stress on rodent models of neurogenesis has long been established, these recent papers have the merit of convincingly extending this concept to human samples. Of note, unlike IL-1 and HMGBI, inflammatory mediators related to cell damage and microglial activation, other “neuropoietic” cytokines such as LIF, CNTF and CT-1 promote NSC self-renewal and progenitor cell division and differentiation in mouse brain, likely through the Janus kinase-signal transducer and activator of the transcription (JAK/STAT) pathway [90].

(e) Chemical mediators from microbioma metabolism: communication between the gut microbiota and CNS also occurs through microbial-derived intermediates, the most relevant being SCFAs [33], tryptophan metabolites [73] and secondary bile acids [91]. Among SCFAs, acetate, propionate, and butyrate are the most abundantly present in the intestinal lumen. These molecules interact directly with enteroendocrine cells, mucosal immune cells, and vagal nerve terminations to propagate bottom-up signalling [92]. Moreover, since they can cross the BBB and bind to brain G protein-coupled receptors, SCFAs can act on both the peripheral and central nervous systems [29], thus exerting their immunomodulatory and anti-inflammatory influence on brain function at multiple levels [85]. More specifically, SCFAs modulate the release of neuropeptides such as serotonin and peptide YY [93], which is potentially relevant for GBA and involved in AN. In addition, SCFAs can affect the integrity of the BBB and regulate the secretion of 5-hydroxytryptamine by enterochromaffin cells, which indirectly impacts emotion and memory. Most relevant to neurogenesis, physiological levels of SCFAs were found to directly stimulate the growth and differentiation of human neural progenitor cells in vitro, a finding corroborated by the increased expression of several neurogenesis-related genes (ATR, BCL2, BID CASP8, CDK2, VEGFA, E2F1, FAS, NDN) in the same experimental setting [94]. These effects on NPCs could be mediated, at least in part, by the SCFA-stimulated endogenous synthesis of serotonin in adult NSC [95]; unfortunately, to the best of our knowledge, this intriguing possibility has yet to be tested.

## 3. Main Modulators of the Microbiota with Impact on Neurogenesis through the GBA

### 3.1. Intrinsic Modulators: Ageing, Oxidative Stress and Inflammation

A large body of literature supports the notion that neurogenesis can be influenced by several pathophysiological conditions including neuroinflammation, oxidative stress, brain injury, adverse early-life stress, and ageing. Relevant to the present article, many of these diverse and often synergistic factors may involve gut microbiota in the causal chain of events leading to impaired proliferation and differentiation of neural precursors in the brain. As an example, in mice deprived of social interactions post waning, changes in gut microbiota composition were found to be associated with learning and memory defects, reduced hippocampal levels of IL-6 and IL-10, and impaired neurogenesis [96]. Other studies have shown that gut microbial composition alterations are frequently associated with neuroinflammation, reduced hippocampal neurogenesis and behaviour disorders such as depression. For instance, Diviccaro et al., 2019 [97] observed in rats treated with finasteride, an inhibitor of the enzyme 5-α-reductase involved in steroid metabolism, a long-term inhibitory effect on neurogenesis accompanied by changes in gut microbiota and an inflammatory state, together with a depressive-like behavioural profile [97]. Furthermore, lower levels of butyrate-producing bacteria in the gut [98] and inflammation of different tissues were found in spontaneously hypertensive rats, suggesting a role for dysbiosis-related cytokine derangement in vascular dysfunction. Interestingly, primary astrocyte cultures from these animals exhibit high basal levels of specific inflammatory cytokines, and butyrate treatment reduces inflammatory markers, again pointing to microbial metabolites as regulators of neuroinflammation [99].

Thus, changes in the composition and metabolic properties of intestinal bacterial communities have consistently been associated with brain inflammation, impaired neurogenesis, and downstream behavioural defects in rodent models of disease; while microbe-triggered neuroinflammation may arguably represent a mechanistic explanation for these findings, however, the actual cause-effect relationships and their molecular underpinnings require further investigation.

**Figure 3:**
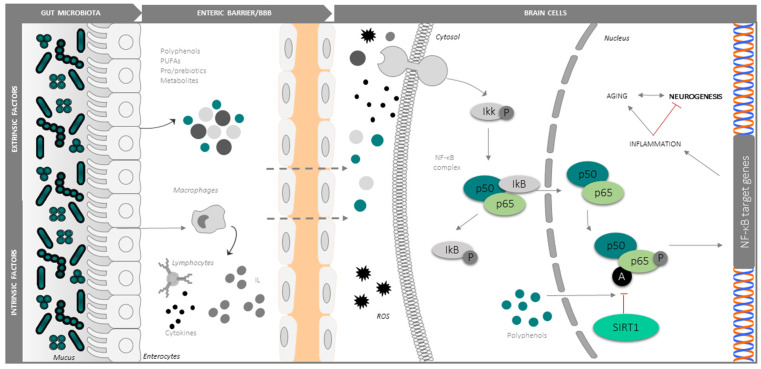
Central role of the SIRT1-NF-kB signalling pathway in neuroinflammation and its modulation by dietary, microbial and immune factors from the intestine. Cytokines, bacterial wall components and ROS activate NF-kB, while polyphenols inhibit proinflammatory signalling via SIRT-1 mediated inhibitory deacetylation of the factor. Both enteric and BBB act as microbe-modifiable checkpoints in the intestine-gut communication. See the text for details.

In agreement with Harman’s free radical/oxidative stress theory [100] of ageing, oxidative stress is alleged as the primary contributor to neurogenic and cognitive decline in the elderly [101]. The rate of oxidative damage increases in senescent tissues, in parallel with a decrease in the efficiency of the antioxidant and repair mechanisms [102,103,104,105]. In a current model, AN decline in the ageing brain is principally caused by oxidative stress and neuroinflammation, which interferes with the pro-neurogenic signalling pathways and factors implicated in self-renewal and differentiation of NSCs, like SIRT1, NF-κB, Notch, Nrf2, and Wnt/β-catenin. Along parallel lines of evidence, important changes in the gut microbiota have been described during ageing. More specifically, age-related gut dysbiosis is deemed a major contributor to the global inflammatory state of the elderly (known as inflammaging). Mechanistically, dysbiosis leads to the release of endotoxins such as lipopolysaccharides and other proinflammatory metabolic products into the systemic circulation via an increase the intestinal permeability, and hence to the CNS through a damaged BBB. In particular, dysbiosis could concur to increase the intestinal and BBB permeability in neurodegenerative disease (e.g., Parkinson’s disease), enhancing the entrance of microbiota-produced substances into the CNS [106] (Figure 3). In ageing and neurodegenerative conditions, microglia exhibits a highly activated phenotype and secretes neurotoxic pro-inflammatory mediators, nitric oxide, cytokines and chemokines (e.g., interleukin IL-1β, IL-6, IL-8, tumor necrosis factor-α (TNFα), transforming growth factor-β), and ROS [107,108]. On the other hand, the “aged”-type gut microbiota is accompanied by increased levels of several cytokines (e.g., IL6, IL-10, TNF-α, TGF-β), the activation of TLR2, NF-κB and mTOR, and decreased levels of cyclin E and CDK2. Specifically, in aged humans and in centenarians, gut bacteria of the phylum *Proteobacteria* exhibit a positive correlation with IL-6 and IL-8, while *Ruminococcus lactaris* correlates with low IL-8 [109]. Thus, even in a small quantity, typical microbial alterations produced in a senescent intestine are associated with gut and brain inflammation [81,110,111].

Several lines of evidence point to the NF-kB signalling pathway [112] and the Notch signalling cascade [113] as central points for the activation of microglia and by extension to neuroinflammation and its downstream pathologic consequences including impaired neurogenesis. NF-κB is expressed both in neurons and glia [114,115], and following stress signals, such as an accumulation of ROS or proinflammatory molecules, the coordinate action of protein kinases that phosphorylate the NF-kB inhibitor IkB [116], and protein deacetylases like SIRT-1 (a potent deacetylator of the lysine 310 of RelA/p65 subunit), modulate the nuclear translocation of the p50/p65 factor and its transcriptional activity at the promoter regions of proinflammatory genes [117,118,119,120,121]. In particular, deacetylation by SIRT1 inhibits the proinflammatory transcriptional program activated by NF-kB, making this circuit attractive for the interventional control of neuroinflammation [122,123,124]. As an example, in c-Rel knockout mice, the unbalanced activity of aberrantly acetylated RelA in the basal ganglia accelerates the senescence of dopaminergic neurons, triggering Parkinson’s-like changes of the substantia nigra with neuroinflammation, and accumulation of alpha-synuclein and iron [125]. Thus, the manipulation of NF-κB acetylation and downstream signalling may be valuable in neurodegenerative disorders, including Alzheimer’s and Parkinson’s disease [126]. Considerable interest exists in developing efficient NF-κB inhibitors for neurodegenerative diseases. Strategies that block molecules upstream of the NF-κB pathway or the associated signalling adapters or those that target the IκB inhibitory proteins have been shown to exhibit significant propensity for systemic and off-target toxicities. Strategies that directly target p65/p50 dimers are likely to regain the homeostasis. Since elevated p65 is highly expressed only in pathologically activated cells, selective targeting of this NF-κB subunit may yield therapeutic drugs with better safety profiles. In recent years, chemical derivatives of natural compounds that inhibit NF-κB have been evaluated for therapeutic potential in neurodegenerative diseases. Mechanistically, the active chemical moiety of many natural compounds such as the diterpenes have been shown to form adducts with select residues of p65, compromising its DNA binding and transactivation ability [127]. Along these lines of investigation, Lim et al. isolated anti-inflammatory *Lactobacillus johnsonii CJLJ103* (LJ) from human fecal microbiota and provided it orally to mice treated with LPS. LJ administration improved LPS-induced memory impairment, inhibited NF-κB activation, enhanced CREB phosphorylation, and increased BDNF expression in the hippocampus [128]. Other studies have shown that gut microbiota alteration by extrinsic stress increases NF-kB activation and TNF-α expression, inducing memory impairment in animal models. Conversely, by restoring gut microbiota composition, an attenuation of the neuroinflammation symptoms in the hippocampus was observed [129]. NF-κB overactivation contributes to the transcriptional signature of ageing [120,130], being relevant for neurogenesis, blocking the reprogramming of aged cells into the pluripotent stem cell (iPSCs) [131,132]. On the contrary, genetic and pharmacological inhibition of the NF-κB signalling pathway prevents age-associated characteristics in different mouse models from accelerated ageing and prevents cell differentiation in favor of a pluripotent state [114,133]. Similarly, the activation of Notch signalling promotes gliogenesis and supports self-renewal of NSCs [134], thus contributing to the maintenance of the undifferentiated state and the active self-renewing growth of NSCs [135]. Notably, the Notch signalling pathway is also involved in the regulation of microglia activation in response to stress situations such as hypoxia by increasing the expression and translocation of intracellular Notch receptor domain (NICD) together with RBP-Jκ and target gene Hes-1 expression, partly through the crosstalk with NF-κB/TLR4/MyD88/TRAF6/pathways. Specifically, Notch inhibition reduced NF-κB/p65 expression and translocation [135]. Intriguingly, Notch and Wnt/β-catenin signalling pathways are essential for the maintenance of intestinal functions and homeostasis [136,137], and are modulated by both microbiota and diet [136].

Relevant to the present article, of the few gastrointestinal tract-derived microbes so far studied, all appear capable of triggering an NF-κB-miRNA-146a signalling pathway to promote neuropathological changes such as amyloidogenesis, apoptosis, inflammatory neurodegeneration, synaptic and neurotropic defects. Furthermore, microbial components secreted in the gut (e.g., neurotoxic exudates, endotoxins and exotoxins, fragilysin, select lipoglycans, lipopolysaccharide LPS, and microRNA-like small non-coding RNAs) affect the entire neural system [138,139]. Therefore the modulation of these factors and their impact on the Wnt/ β-catenin, Notch and NF-κB signalling may serve at multiple levels of the GBA as a prospective target for inhibiting microglia activation and inflammation, with the aim of improving neurogenesis in neurodegeneration and ageing [113].

### 3.2. Extrinsic Modulators

The microbiota-GBA-neurogenesis circuit is amenable to control by extrinsic modulators that could be exploited therapeutically. These include lifestyle, physical exercise, and above all diet modifications, for example through caloric restriction or diet component adjustments.

#### 3.2.1. Antioxidants and Anti-Inflammatories: Polyphenols

Several studies indicate that dietary antioxidants can attenuate oxidative damage and preserve cognitive function [140] in the ageing brain by suppressing the expression of senescence-related genes [140,141]. A decrease in AN accompanies age-related cognitive decline and correlates with reduced concentrations of antioxidants in both serum [142] and brain tissue [142], thus providing a strong rationale for antioxidant supplementation [143]. Polyphenols (e.g., resveratrol) are plant-derived natural compounds endowed with antioxidant and anti-inflammatory properties and are able to cross the enteric and BBB [144,145,146]. The antioxidant activities of polyphenols are manifold: they (a) directly quench ROS [147], (b) inhibit the major ROS-forming enzymes, including monoamine oxidase or xanthine oxidase [148], (c) chelate metal ions (iron and copper) involved in ROS reactions [149], and (d) regulate the redox metal homeostasis and prevent the metal deposition and neurotoxicity, with important implications for neurodegenerative diseases as dementia, Alzheimer’s disease and Parkinson’s disease [150]. Most important, polyphenols can modulate signalling pathways and factors relevant to neurogenesis, including SIRT1, NF-κB, Nrf2 and Wnt/β-catenin [46], and are under active investigation as adjuvants in neurodegenerative disorders with impaired AN. At the opposite end of the GBA, polyphenols modify microbiota composition by favouring the expansion of some bacterial species and inhibiting others. Furthermore, gut bacteria can metabolize polyphenols into neurotransmitters and bioactive metabolites with pro-survival and anti-inflammatory effects on neurons [85]. The effect of polyphenols on intestinal microbes and their recently highlighted importance as intermediate substrates for microbial synthesis of bioactive factors adds a new dimension to the long described neuroprotective and proneurogenic action of these natural compounds, opening them to further research aimed at microbiota and GBA-centered interventions against neurodegeneration and brain ageing symptoms [93].

#### 3.2.2. Polyunsaturated Fatty Acids (PUFAs)

PUFAs are essential unsaturated fatty acids with more than one double bond (C=C). They are important nutrients that must be obtained primarily from the diet or from supplements as mammals cannot synthesize them de novo. Long chain PUFAs can be divided into two main biologically important groups: n-6 PUFAs (omega-6) and n-3 PUFAs (omega-3), with their first double bond at C6 or C3 counting from the methyl C, respectively. Besides, Linoleic acid (n-6 PUFA) and α-linolenic acid (n-3 PUFA) are essential fatty acids, as humans can’t produce them and are the precursor of other important PUFAs [151,152]. Among the nutritionally important PUFAs, α-linolenic acid, eicosapentaenoic acid and docosahexaenoic acid are highly concentrated in the brain and have antioxidant, anti-inflammatory and antiapoptotic effects [153]. Relevant to the present article, dietary PUFAs have a significant impact on the intestinal microbial ecosystem [154]. In mice engineered to overproduce n-6 PUFAs and to increase the n-6 to n-3 ratio, signs of systemic inflammation have been reported in association with evidence of intestinal dysbiosis (increased Proteobacteria while reduced Bacteroides and Actinobacteria) and abnormal gut permeability. In the same study, instead, transgenic enhancement of n-3 PUFAs tissue content inhibited LPS induced inflammation, preserved and stimulated the growth of Bifidobacterium, and preserved the intestinal barrier [155]. While the authors focused their attention on metabolic endotoxemia as the main dysbiosis-associated disorder in their transgenic animals, this work outlines a possible PUFA-microbiota-brain signalling circuitry to be further explored.

#### 3.2.3. Probiotics/Prebiotics

Natural bioactive molecules such as probiotics and prebiotics can modify the gut microbiota composition and are therefore attracting increasing interest for the adjuvant treatment of an array of dysbiosis-associated disorders, from intestinal inflammation to autism spectrum disorders [156]. According to a criteria established by the WHO, a probiotic is defined as a live organism which provides a benefit to the host when provided in adequate quantities [157]. Numerous organisms meet the criteria, but the leading ones are *S. boulardii*, the Gram-negative *E. coli* strain Nissle 1917, various lactic-acid-producing lactobacilli strains, and a number of bifidobacteria strains (for a list see Table 1 of [158]). Instead, prebiotics are defined by the International Scientific Association of Probiotics and Prebiotics as a selectively fermented ingredient that results in specific changes in the composition and/or activity of the gastrointestinal microbiota, thus conferring benefits upon host health [159]. The majority of them are a subset of carbohydrate groups, mostly oligosaccharide carbohydrates such as fructo-oligosaccharides and galacto-oligosaccharides. The effects of prebiotics on human health are mediated through their degradation products by microorganisms; fermentation of prebiotics by gut microbiota produces SCFAs, including lactic acid, propionic acid, and butyric acid. This last, for example, influences intestinal epithelial development. Since SCFAs can diffuse to blood circulation through enterocytes, prebiotics have the ability to affect not only the gastrointestinal tract but also distant site organs such as the brain [160], for example by the vagus nerve [161]. Some prebiotics, such as fructo-oligosaccharides and galacto-oligosaccharides, have regulatory effects on BDNF, neurotransmitters (e.g., d-serine), and synaptic proteins (e.g., synaptophysin and NMDA receptor subunits) [162,163]. Other beneficial effects of probiotics involve the maintenance of immune homeostasis through the block of pathogen growth via the release of lactic acid, the inhibition of systemic immune responses, and the preservation of intestinal barrier integrity [164,165]. Relevant to the present article, the administration of probiotics has been reported to impact cognition and behaviour; for example the combination of *Lactobacillus helveticus* and *Bifidobacterium longum* decreases anxiety in rats and humans [166]. In aged rats, the administration of VSL#3, a probiotic mixture, reduces inflammation by reducing IL-10 protein expression and contributes to the increase BDNF and synapsin mRNA in the hippocampus [167]. In a study investigating the effect of *Lactobacillus* strains on cognitive decline in ageing-accelerated mice, a diet supplemented with *Lactobacillus paracasei* K71 improved cognitive performance probably through an upregulation of hippocampal BDNF expression [168]. In rats chronically fed with a chronic high-fat diet, the supplementation with *Lactobacillus paracasei* HII01was able to increase hippocampal plasticity and attenuate brain mitochondrial dysfunction and microglial activation [169]. The administration of SLAB51, a probiotic formulation of lactic acid bacteria and bifidobacteria, activates SIRT1 and promotes antioxidant and neuroprotective effects in a mouse model of Alzheimer’s disease [170]. All together these results seem to indicate that probiotics may be beneficial in maintaining brain function; although a solid link with neurogenesis is still missing, the generation of SCFAs modulation of oxidative stress and inflammation and the upregulation of hippocampal BDNF all represent potential “pro-neurogenic” activities of probiotics and prebiotics to be further investigated in the appropriate experimental models [61].

#### 3.2.4. Physical Exercise

Physical exercise and the gut microbiome have been independently described to prevent age-related global brain atrophy and both increase brain volume in the frontal lobes and left superior temporal lobe, which are important for cognition, attention and memory [171]. Data suggest that within the hippocampus, the dentate gyrus is susceptible to exercise intervention, with an increase in exercise-related neurogenesis. Specifically in humans, aerobic exercise was linked with the upregulation of serum levels of BDNF (a mediator of neurogenesis in the dentate gyrus) and with greater hippocampal volume and a subsequent decrease in psychological disorders (e.g., depression, anxiety) [172]. Moderate-intensity aerobic exercise training has also been described to improve mood and brain functional activation in older adults aged 60–79 years [173,174]. To date, specific mechanisms linking exercise with GBA and the brain have not been described; however, exercise appears to alter the gut biodiversity in both quantitative and qualitative ways [175]. Aerobic exercise has been posited to increase microbiome diversity, alter functional metabolism and modify the by-products released by intestinal bacteria [176]. More specifically, it has been demonstrated that aerobic exercise improves microbiome diversity in humans increasing genera of the *Firmicutes* phylum (specifically, *Faecalibacterium prausnitzii* and species from the genus *Oscillospira, Lachnospira*, and *Coprococcus*), which produce an enriched profile of SCFAs [177]. Some of the identified SFCAs (acetate, propionate, and butyrate, which are produced by gut microbes from protein, fibers, and nondigestible starches [178]), are essential to neuro-immunoendocrine regulation. For example, SCFAs influence neuroinflammation by affecting glial cell morphology and function as well as by modulating the levels of neurotrophic factors, increasing neurogenesis, contributing to the biosynthesis of serotonin, and improving neuronal homeostasis and function [179]. Additionally, exercise-induced changes in intestinal microbiota may improve the function of the gut-vascular barrier (GVD), possibly by increasing the intestinal content of the bile acid analogue and farnesoid X receptor agonist obeticholic acid. In view of the recently discovered functional linkage between GVD with the BBB [180], a positive impact of these changes on brain function and in particular on neurogenic activities in BBB-supported niches could easily be envisaged [181]. At the moment, however, the possibility that this gut-centered circuitry contributes to neurogenesis regulation by physical exercise remains speculative.

## 4. Future Challenges and Conclusions

Although much evidence points to a pivotal role for intestinal microbiota and bacteria-derived metabolites in the gut-brain communication axis and in particular in the modulation of AN, the consolidation of this critical knowledge and its possible translation to the clinical practice still face a number of daunting challenges. The first challenge deals with the lack of reliable approaches to reproducibly assess AN in humans, and above all in living individuals. This limitation applies to the entire AN research field, to the point that it was not until recently that the question as to whether AN is relevant to human health has begun to be settled [182,183]. Even in the most rigorous studies, adult neurogenesis is assessed by the immuno-histochemical detection of putative markers for neural precursors and immature neurons whose reliability is mainly inferred from animal models. Additionally, since no prospective labelling is possible in human samples, uncertainty exists as to whether neuroblasts/immature neurons will fully differentiate and integrate long term in the preexisting neural network [184]. Research on human adult neurogenesis and its modulation by endogenous and exogenous factors including nutrition, microbiota and lifestyle would benefit greatly from the possibility of detecting and measuring the process non-invasively. In this respect, initial enthusiasm resulting from the possibility (reported some 15 years ago) of detecting NPCs by nuclear resonance spectrometry brain imaging in vivo [185] has not been followed up by convincing evidence. The development of biochemical markers and/or imaging approaches to quantify new neurons/neural precursors and evaluate their functionality, coupled with advances in metagenomics and metabolomics techniques for the individualized characterization of intestinal microflora (for which this article is concerned) will hopefully help overcome these seemingly insurmountable obstacles.

A second challenge comes from the elucidation of the specific molecular mechanisms underlying the influence of gut microbiota on neurogenesis, even in the experimental (i.e., animal) setting. In fact, many of the currently hypothesized mechanism are chains of logical connections between phenomena (i.e., microbiota regulation by dietary factors → microbial control of neuroinflammation → modulation of neurogenesis by inflammaging) occurring at the two ends of the GBA, without any mechanistic linkage being truly demonstrated. Related to this, distinguishing direct (i.e., affecting neuronal precursors) and indirect (i.e., resulting from systemic neurohormonal or even behavioural response) effects of intestinal bacteria on neurogenesis will likely require further theoretical and methodological efforts.

Specifical future lines of research could clarify how selected molecular targets involved in neurogenesis, such as NF-κB and SIRT1, are susceptible to modification by diet components or microbiome metabolites, as well as how multiple molecular pathways act in a synergic crosstalk. Overall, an integrative approach appears advisable based on the study of the topic from different perspectives. More basic research on animal and cell culture models is needed to characterize compounds potentially active on neurogenesis and gut microbiota in terms of dosing, bioassimilation and the combinatorial effect, both in physiological and pathological settings. On the other hand, more clinical research is needed (with the limitations acknowledged above), to clinically test the efficacy of promising molecules, particularly antioxidants and anti-inflammatory natural compounds of microbial origin, for their neuroprotective and possibly neurogenic effects. The ultimate goal (or hope) of this future research will be to establish the basis for the discovery of advanced therapeutics and the identification of novel biomarkers, which may help with early intervention and the prevention of brain disorders in which neurogenesis likely plays an essential role.

## Data Availability

Not applicable.

## Abbreviations

AN	adult neurogenesis
BBB	blood brain barrier
BDNF	brain-derived neurotrophic factor
BrdU	bromo-2-deoxyuridine
CREB	cAMP response element-binding protein
GBA	gut-brain axis
GVD	gut-vascular barrier
CNS	central nervous system
GF	germ-free
HPA	hypothalamic–pituitary–adrenal axis
IGF	insulin-like growth factor
IL	interleukin
NPCs	neural progenitor cells
NSCs	neural stem cells
LJ	*Lactobacillus johnsonii CJLJ103*
PUFAs	Polyunsaturated fatty acids
ROS	reactive oxygen species
SEZ	subependymal zone
SCFAs	short-chain fatty acids
TNFα	tumor necrosis factor-α
TLR2	toll-like receptor
